# Editorial: Can traditional Chinese medicines affect endocrine diseases via effects on the intestinal flora?

**DOI:** 10.3389/fendo.2023.1193396

**Published:** 2023-05-16

**Authors:** Xinhua Shu, Guang Chen, Zhoujin Tan

**Affiliations:** ^1^ Pu Ai Medical School, Shaoyang University, Shaoyang, China; ^2^ Department of Biological and Biomedical Sciences, Glasgow Caledonian University, Glasgow, United Kingdom; ^3^ Department of Vision Science, Glasgow Caledonian University, Glasgow, United Kingdom; ^4^ Department of Integrated Traditional Chinese and Western Medicine, Tongji Hospital, Tongji Medical College, Huazhong University of Science and Technology, Wuhan, China; ^5^ College of Chinese Medicine, Hunan University of Chinese Medicine, Changsha, Hunan, China

**Keywords:** endocrine disorder, traditional Chinese medicine, gut microbiota, therapy, molecular mechanisms

Human gut microbiota is a complex and dynamic ecosystem, containing over 1500 microbial species and comprising appropriately 9.9 million microbial genes (1, 2). The diversity of human gut microbiota rapidly expands from infancy to early childhood, then the expansion slows down during preadolescence; the diversity remains quite stable in adulthood but gradually decreases with ageing (3). Various factors, including host genetic features and environmental influences, regulate gut microbiota diversity, among which diet is considered to be a predominant factor. Gut microbiota has multiple functions, including maintenance of the structural integrity of the gut mucosal barrier; suppression of pathogen overgrowth; regulation of host immune response, endocrine and neurologic signalling pathways; metabolism of host nutrients, bile salts and xenobiotic substances; elimination of exogenous toxins; and synthesis of neurotransmitters, vitamins and other uncharacterized compounds (3). Disruption of gut microbiota diversity and function, termed as dysbiosis, is associated with a wide range of human diseases, such as endocrine and neurodegenerative diseases (3).

Traditional Chinese medicine (TCM) has been widely used for the prevention and treatment of human disease in China and other Asian countries over thousands of years (4). Currently, TCM is recognized as the most important alternative to Western medicine. TCM formulas contain a variety of functional compounds, particularly polysaccharides and polyphenols. Oral administration of TCM leads directly or indirectly to interaction between TCM and gut microbiota. TCM bioactive ingredients can regulate the diversity and function of gut microbiota, while enzymes produced by gut microbiota participate in the metabolization of TCM. The resultant metabolites have shown therapeutic effects in a wide range of diseases, including endocrine conditions (4). However, the underlying mechanisms are not fully understood. The current Research Topic aims to collect recent progress in elucidating the functional interplay between TCM and gut microbiota in treating endocrine diseases. We welcomed original research and review papers concerning the molecular mechanisms and protective effects of TCM-mediated treatments in animal models and/or patients with endocrine diseases. We collected 6 review articles and 14 original research papers.

Diabetes mellitus is the commonest endocrine disease and is characterized by deficient insulin production or insulin resistance. There are two types of diabetes: type 1 and type 2 diabetes. In the former, destruction of pancreatic beta cells results in reduced insulin production; in the latter, less insulin is produced or the insulin is less effective. Gut microbiota also plays an important role in diabetes. TCM treatment has been shown to regulate gut microbiota composition and function. Su et al. discussed the correlation of gut microbiota with type 2 diabetes, and the effects of individual Chinese herb ingredients (polysaccharides, saponins, polyphenols, alkaloids and flavonoids) and medicinal plant extracts (licorice and *Sargassum fusiforme*) on gut microbiota balance in diabetic rodent models. The authors also give examples of TCM formulas, including Huang-Lian-Jie-Du decoction, Xie-Xin decoction, Ge-Gen-Qin-Lian decoction, Pi-Dan-Jian-Qing decoction, Shen-Ling-Bai-Zhu powder, Shen-Qi compound, Liu-Wei-Di-Huang pills, San-Huang-Yi-Shen capsule and Tang-Nai-Kang, which have been show to effectively counteract diabetes in diabetic rodent models, at least in part *via* positive regulation of gut microbiota. He et al. further discussed the protective function of berberine, a member of the alkaloid group, against type 2 diabetes. The antidiabetic capacity of berberine is at least in part mediated by the regulation of gut microbiota. Berberine can inhibit opportunistic pathogen growth, induce death of harmful gut microbiota, and promote proliferation of beneficial bacteria. Berberine treatment can increase synthesis of short chain fatty acid (SCFA) in the intestine and help to maintain the integrity and function of the intestinal barrier. Furthermore, berberine can enhance metabolism of bile acid and amino acids in the intestine. The authors also updated the literature related to the protective effects of berberine against diabetes-associated metabolic disorders in clinical trials. Li et al. reported that Huang Lian extract, which contains multiple functional compounds, including berberine, had protective effects against type 2 diabetes *via* a shifting of the composition of the gut microbiota by enriching bacterial species associated with bile acid metabolism. In addition, *Inonotus obliguus* extract and individual compound (chlorogenic acid) demonstrated similar protection against diabetes in db/db mice by regulating gut microbiota composition (Yan et al.; Ye et al.). Additionally, Shi et al. and Huang et al. demonstrated that salidroside (the predominant active component isolated from Rhodiola) and Zuogui Jiangtang Shuxin formula alleviated, respectively, diabetic cardiomyopathy and improved myocardial function in diabetic mouse models, possibly in part mediated by remodelling the structure of gut microbiota. Obesity is closely associated with type 2 diabetes. Extensive evidence suggests that obesity is linked to disruption of gut microbiota and impairment of gut barrier function. Li et al. reviewed literature concerning the regulation of gut microbiota by TCM in obesity. TCM ingredients can enhance the abundance of gut microbiota and the generation of beneficial metabolites, regulate lipid metabolism and decrease fat accumulation, and counteract high-fat-induced inflammation. Hu reported that *Grifola frondosa* powder had anti-obesity capacity by regulating the structure and composition of gut microbiota and increasing production of SCFA.

Non-alcoholic fatty liver disease (NAFLD) is the most common chronic liver disease and is characterized by the presence of steatosis in the absence of over-consumption of alcohol. NAFLD is a complex disease, associated with other metabolic disorders such as obesity, diabetes and hyperlipidemia (5). Data from NAFLD patients and rodent models has shown dysbiosis is associated with NAFLD (Guo et al.). Individual TCM compounds or TCM decoctions can repair intestinal barrier function, inhibit growth of pathogenic bacteria in the intestine, suppress inflammation, and decrease lipid deposition in the liver, leading to alleviated liver steatosis in NAFLD animal models (Guo et al.). Lv et al. reported the protective effect of Qingrequzhuo capsule against nonalcoholic steatohepatitis (NASH), a type of NAFLD, in mice fed with methionine and choline deficient diet. The diversity of gut microbiota was decreased in NASH mice; Qingrequzhuo treatment reversed the effect. Colonic permeability was significantly reduced in Qingrequzhuo-treated NASH mice, compared to untreated NASH mice. Qingrequzhuo treatment also downregulated expression of proinflammatory cytokines in the liver of NASH mice *via* inactivation of the TLR4/NF-kB signalling pathway (Lv et al.). In a clinical trial, Hui et al. assessed the beneficial effect of a ‘spleen-strengthening’ and ‘liver-draining’ herbal formula in NAFLD patients. The authors found that the formula improved liver function and glycolipid metabolism, and alleviated fatigue symptom. These protective effects of this formula were mediated by regulating the disturbance of gut microbiota in NAFLD patients (Hui et al.). Additionally, Liu et al. demonstrated that a combination of polysaccharides from *Astragalus membranaceus* and *Hippophae rhamnoides* alleviated liver pathology and repaired disturbance of gut microbiota in mice with alcoholic fatty liver disease.

This Research Topic also collected papers related to other endocrine-associated disorders. Liu et al. reviewed the literature concerning the relation between gut microbiota and polycystic ovary syndrome (PCOS), a common reproductive endocrine disorder. PCOS is associated with hyperandrogenemia, insulin resistance, obesity, and chronic inflammation. It is well documented that gut microbiota regulates these PCOS-associated risk factors, so indicating that gut microbiota has a direct and/or indirect relationship with PCOS (Liu et al.). Ping et al. investigated the effect of *Atractylodis macrocephalae rhizoma* polysaccharide on mammary gland hyperplasia and found that polysaccharide treatment exhibited therapeutic effects against mammary gland hyperplasia in rats by decreasing the production of estradiol and prolactin, increasing the progesterone level, and regulating the diversity and abundance of gut flora; the same group also developed microneedles loaded with aqueous extract of *Atractylodis macrocephalae rhizome* and found that the microneedles had similar protective effects on mammary gland hyperplasia and on gut microbiota as the polysaccharide (Ping et al.). Xia et al. evaluated the effect of Si-Jun-Zi decoction (SJZD) on diarrhoea-predominant irritable bowel syndrome (IBS-D) by co-culturing SJZD with intestinal microbiota from healthy controls and IBS-D patients. The authors found that co-culture with SJZD reversed IBS-D associated dysbiosis and regulated the production of bacterial metabolites that are functionally linked to IBS-D-associated neurotransmitters. Shang et al. compared the difference in gut microbiota in patients with metabolic syndromes associated with TCM: specifically, qi-yin deficiency syndrome (QYDS) or phlegm-dampness syndrome (PDS). QYDS refers to the damage of both Yang Qi and Yin liquid in the body and is characterized by shortness of breath, chest pain, spontaneous sweating, and fatigue; PDS refers to spleen deficiency and formation of phlegm overtime with main symptoms of coughing, phlegm accumulation and chest tightness. The authors found that there was significant difference in gut microbiota structure between the two patient groups. The authors also noticed that there was correlation between gut microbiota genera and clinical phenotypes in both patient groups. Aging is a complex metabolic process that is associated with dynamic changes in host gut microbiota. Liu et al. showed an anti-aging capacity of *Dubosiella newyorkensis* in aged mice by inhibiting oxidative stress and inflammation and promoting growth of beneficial genus.

In summary, the articles in this Research Topic collection provide novel insights into the functions and underlying mechanisms of TCM in treating endocrine disorders. The Research Topic will contribute to the development of new TCM treatment for a wide range of endocrine disorders and strengthen the dissemination of TCM knowledge to the international community.

## Author contributions

XS wrote the editorial. ZT and GC read and revised the editorial. All authors contributed to the article and approved the submitted version.
